# Supplementary figures and images for: A study on substitution characteristics and competitive performance of Chinese super league teams under the five-substitution rule
Source: PLoS One. 2025 May 12;20(5):e0322241. doi: 10.1371/journal.pone.0322241 (PMC12068657; doi:10.1371/journal.pone.0322241)

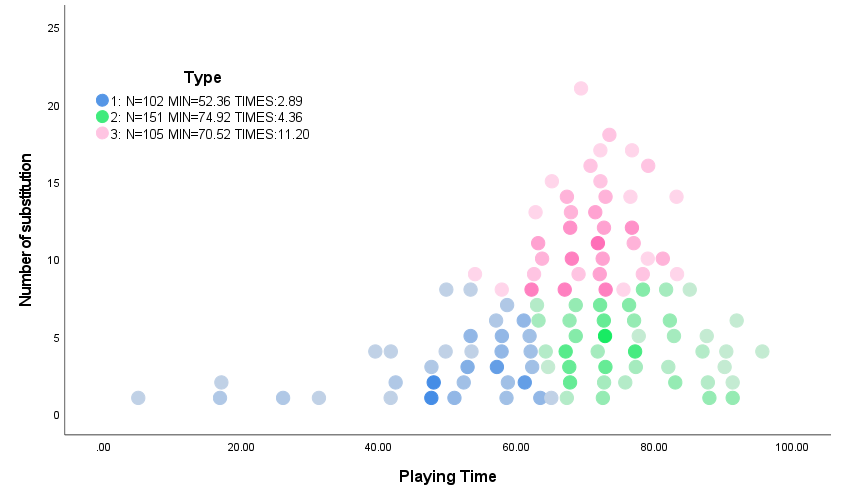

Supplement: S1 Fig — primarily categorizes players based on their playing time and the number of substitutions, including both substituted and replaced players. (ZIP) [file pone.0322241.s002.zip › S1_fig/The illustration of clustering Replaced Players.docx]

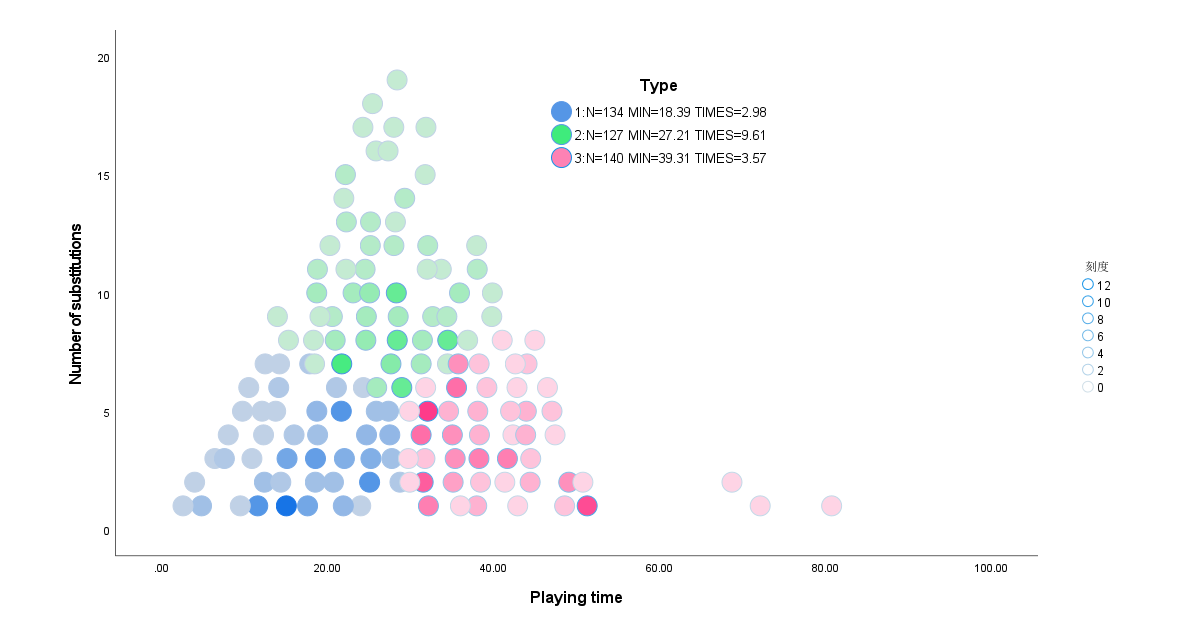

Supplement: S1 Fig — primarily categorizes players based on their playing time and the number of substitutions, including both substituted and replaced players. (ZIP) [file pone.0322241.s002.zip › S1_fig/The illustration of clustering Substitute Players.docx]
