# Supplementary material for: A study on substitution characteristics and competitive performance of Chinese super league teams under the five-substitution rule
Source: PLoS One. 2025 May 12;20(5):e0322241. doi: 10.1371/journal.pone.0322241 (PMC12068657; doi:10.1371/journal.pone.0322241)
Supplement: Attachment — Submitted filename: Response to Reviewers.docx [file pone.0322241.s008.docx]

Dear Editor Gardasevic,

Thank you for giving us the opportunity to submit a revised draft of our manuscript titled "A Study on Substitution Characteristics and Competitive Performance of Chinese Super League Teams under the Five-Substitution Rule" [PONE-D-25-03456] to PLOS ONE. We appreciate the time and effort that you and the reviewers have dedicated to providing valuable feedback on our manuscript. We have incorporated changes that reflect all the suggestions provided by the reviewers. All changes are highlighted in red in the revised manuscript. Please see below for our point-by-point responses to the reviewers' comments. We hope that our work can be improved again.

**Response to Comments of Reviewer *#*1：**

**Comment 1**：It is not clear from the abstract which sport it is. That is unacceptable. The study is interesting. It provides information that an adequate substitution strategy can improve team performance.

**Response to comment 1:**We sincerely appreciate your careful review and your interest in our research topic. Regarding your comment on the lack of clarity in distinguishing the sport in the abstract, we acknowledge that this was an unacceptable oversight on our part. We sincerely apologize for this mistake. Based on your suggestion, we have carefully revised and supplemented the abstract by explicitly specifying that our study focuses on soccer. This modification ensures that readers can clearly understand the context of our research. We genuinely hope that these improvements enhance the clarity and quality of the abstract. Thank you again for your valuable feedback.

**Comment 2**：I would recommend that the proofreader look at this text. I think the academic style of English could be improved. There are too many complex sentences that make it difficult to understand. Like for example this one: "A stable substitution structure centered around core players is conducive to better seasonal performance." That could be said more simply. There are many such sentences throughout the text as well.

**Response to comment 2:**Thank you for bringing these concerns to our attention. We sincerely apologize for any confusion caused by the previous version of the manuscript. We have taken significant efforts to enhance both the language quality and overall readability. We also sought the assistance of native English speakers to refine the language for improved precision and cohesion.

Additionally, we have conducted a thorough revision of the abstract, introduction, and discussion sections, refining inappropriate expressions and improving the overall structure and language. We have reorganized the logic and terminology to enhance the professionalism of the writing. Furthermore, we have simplified complex sentences by removing redundant elements to make the text more concise and readable. For example, we revised "A stable substitution structure centered around core players is conducive to better seasonal performance." to "a substitution strategy centered around some soccer players contributes to better seasonal performance. " *(See Abstract).* Similar modifications have been made throughout the manuscript to improve sentence structure and grammar. All revised sentences have been highlighted in red for your convenience in reviewing these changes.

**Comment 3**：A paragraph on Limitations of the study should be added to the Discussion. Also a paragraph with recommendations for coaches. Something like this: Coaches can optimize their substitution strategy by...

**Response to comment 3:**We are grateful for your insightful comment, which allowed us to incorporate an important discussion on the limitations of this study and practical recommendations for coaches. Acknowledging research limitations and providing guidance for soccer coaches in substitution strategies is highly meaningful, as it not only highlights areas that require further investigation but also offers practical applications for coaching decisions. We sincerely apologize for the initial omission of this crucial aspect. Based on your recommendation, we have added the following content to the discussion section:

Despite providing valuable insights into substitution strategies in CSL teams, this study has several limitations. First, the analysis is based on data from a single season, which may limit the generalizability of the findings. Second, this study does not fully account for goal difference and match context, all of which can significantly influence substitution decisions.Thus, further research is needed to examine how substitution strategies interact with goal difference, match phase, and opposition strength, while multi-season and cross-league analyses, along with player workload metrics, could enhance understanding and predictive accuracy of substitution decisions.

These findings may be useful for coaches when they deciding substitute or replaced players. Coaches can optimize their substitution strategy by considering players’ importance within the substitution network and their performance. A highly centralized substitution network may contribute to greater team seasonal performance.Therefore, maintaining consistent substitute or replaced players in critical positions to ensure that they can seamlessly integrate into the tactical system could be beneficial**.***(See Discussion)*

Thank you once again for your constructive feedback. We truly appreciate your time and effort in reviewing our manuscript.

**Response to Comments of Reviewer *#2*：**

**Comment 1**：Very current and interesting topic. Especially since there is little research on this issue, and the load and recovery of athletes in modern competitive calendars can be decisive in achieving results. The results of the study are clearly presented, the methodology is respected. The language is understandable. The discussion is very detailed.

**Response to comment 1:**We sincerely appreciate your positive feedback on our study. We are delighted that you found the topic both current and interesting, particularly given the limited research on this issue. The impact of soccer substitute within modern competitive calendars is indeed a crucial factor in the athlete load and recovery , and we are pleased that our study contributes to this important area of research.

We are also grateful for your acknowledgment of the clarity of our results presentation, the rigor of our methodology, and the comprehensiveness of our discussion. Ensuring methodological accuracy and providing a detailed interpretation of findings were key priorities in our work, and it is encouraging to receive such recognition.

Think you and best regards.

Yours sincerely

Chen Tong

Corresponding author:

Name: Chen Liang

E-mail: cullencl@126.com
