# Supplementary material for: A study on substitution characteristics and competitive performance of Chinese super league teams under the five-substitution rule
Source: PLoS One. 2025 May 12;20(5):e0322241. doi: 10.1371/journal.pone.0322241 (PMC12068657; doi:10.1371/journal.pone.0322241)
Supplement: Attachment — Submitted filename: Response_to_Reviewers_auresp_2.docx [file pone.0322241.s009.docx]

Dear Dr. Travassos and Reviewers,

We would like to express our sincere gratitude for your thoughtful and constructive comments on our manuscript titled "A Study on Substitution Characteristics and Competitive Performance of Chinese Super League Teams under the Five-Substitution Rule" [PONE-D-25-03456]. We have carefully considered all feedback and made the appropriate revisions to improve the manuscript. Below, we provide a detailed response to each of the reviewers' comments and the editorial suggestions.

**Response to Comment of Editor:**

"The authors should revise the manuscript according to reviewers' suggestions. Additionally, the authors should improve the description of the concepts: substitution networks, substitution links, and further explain the meaning of the concept 'Higher density indicates closer substitution relationships among players, while lower density reflects looser connections.' The point is, what does 'closer substitution relationship' mean?"

**Response:**We greatly appreciate the editor’s insightful comments. To improve clarity and provide a deeper understanding of these key concepts, we have made the following revisions:

Substitution Networks: We have clarified that substitution networks is a social network constructed based on the substitution patterns of a team during matches where nodes represent players (substitutes or players substituted off) and edges represent substitution relationships during the match. These relationships capture the dynamics of player changes, which reflect substitution strategies.

Substitution Links: We further define substitution links as the connections between the player substituted off and the player who replaces them. We also explain how these links are formed and analyzed in our model.

Density of Substitution Networks: We have expanded on the explanation of network density. A higher density indicates that players on the team have a diverse set of substitution interactions, showing that the team’s substitution decisions involve a wider range of player combinations. A lower density, on the other hand, suggests that substitution decisions tend to involve only a small set of players, reflecting less variability in the team’s substitution strategy.

These revisions are included in Section Methods of the manuscript, where we now provide a more comprehensive explanation of these terms to ensure the concepts are fully understood by readers.

**Response to Comments of Reviewer *#*1：**

We sincerely appreciate your positive feedback and recognition of our improvements. Your comments have been instrumental in enhancing the clarity and quality of our manuscript.

**Response to Comments of Reviewer *#2*：**

Thank you very much for your encouraging words and constructive feedback. We are grateful for your support and recognition of our efforts to improve the manuscript.

Think you and best regards.

Yours sincerely

Chen Tong

Corresponding author:

Name: Chen Liang

E-mail: cullencl@126.com
